# GPT-based normative models of brain sMRI correlate with dimensional psychopathology

**DOI:** 10.1162/imag_a_00204

**Published:** 2024-06-26

**Authors:** Sergio Leonardo Mendes, Walter Hugo Lopez Pinaya, Pedro Mario Pan, Ary Gadelha, Sintia Belangero, Andrea Parolin Jackowski, Luis Augusto Rohde, Euripedes Constantino Miguel, João Ricardo Sato

**Affiliations:** Center of Mathematics, Computing, and Cognition, Universidade Federal do ABC, São Paulo - SP, Brazil; Department of Biomedical Engineering, King’s College London, London, United Kingdom; Escola Paulista de Medicina, Universidade Federal de São Paulo, São Paulo - SP, Brazil; Department of Education, ICT and Learning, Østfold University College, Halden, Norway; ADHD Outpatient Program & Developmental Psychiatry Program, Hospital de Clinicas de Porto Alegre, Federal University of Rio Grande do Sul, Brazil; Medical Council UNIFAJ and UNIMAX, Indaiatuba, Brazil; National Institute of Developmental Psychiatry & National Center for Innovation and Research in Mental Health, São Paulo – SP, Brazil; Department and Institute of Psychiatry, Universidade de São Paulo (USP), São Paulo - SP, Brazil; Big Data Section, Hospital Israelita Albert Einstein, São Paulo - SP, Brazil

**Keywords:** children, brain structural MRI, GPT models, child behavior checklist, autism spectrum disorder, attention deficit hyperactivity disorder

## Abstract

Generative Pre-trained Transformer (GPT) models have been widely used for language tasks with surprising results. Furthermore, neuroimaging studies using deep generative normative modeling show promise in detecting brain abnormalities from brain structural MRI (sMRI). Meanwhile, psychiatric disorders are typically diagnosed through clinical assessment, which is particularly challenging in children and adolescents who present early symptoms or are in the early stages of the disease. Brain biomarkers research may contribute to the complex task of disentangling typical neurodevelopment from emergent psychiatric disorders. Here, we investigate whether a GPT-based normative architecture can detect psychiatric symptoms and disorders from brain sMRI of youths. The studied datasets contain measures of dimensional psychopathology: Brazilian High-Risk Cohort Study (BHRCS,*n = *737) and Adolescent Brain Cognitive Development (ABCD,*n = *11,031), and scores and diagnostic of psychiatric disorders: Attention Deficit Hyperactivity Disorder (ADHD-200,*n = *922) and Autism Brain Imaging Data Exchange II (ABIDE-II,*n = *580). We examined the associations of all brain regions with: the Child Behavior Checklist (CBCL) symptom groups, ADHD scores, and Autism Spectrum Disorder (ASD) diagnosis. Results showed the whole-brain typicality likelihood as correlated with social problems (ABCD test set) and ASD diagnosis (ABIDE-II dataset). Analysis by brain regions linked different areas to several CBCL scales, ADHD scores, and ASD diagnostic. This is the first successful study assessing all dimensional groups of CBCL symptoms, from all brain regions, based exclusively on sMRI. The normative models based on GPT are promising to investigate the gap between the phenotypes of psychiatric conditions and their neurobiological substrates.

## 1 Introduction

One of the big challenges psychiatrists face is how to incorporate biological measures in diagnosing mental disorders (Cuthbert & Insel, 2010;Scarpazza et al., 2020). Besides some advances (Drysdale et al., 2017), most psychiatric disorders are assessed only by clinical interviews (American Psychiatric Association, 2013;Sato et al., 2017). The early identification of mental health issues is even more difficult. Investigations indicate that less than a fifth of the American youths experiencing symptoms that qualify them for a psychiatric diagnosis are typically identified (Levitt et al., 2007). The time window of childhood and adolescence is of great importance in the development of psychiatric disorders (American Psychiatric Association, 2013). In these age groups, the investigation of brain markers could provide information regarding the pathological mechanisms related to the nature of these diseases. Moreover, the investigation of objective brain markers may contribute to the complex task of disentangling typical neurodevelopment from emergent psychiatric disorders.

Attempts to characterize brains’ structural signatures of psychiatric disorders have shown that these conditions are highly heterogeneous (Cannon & Keller, 2006;Cicchetti & Rogosch, 1996;Marín, 2016). For instance, similar etiopathological mechanisms may converge to the same symptom in different patients (Cannon & Keller, 2006;Cicchetti & Rogosch, 1996). Conversely, several risk factors can result in different clinical phenotypes for distinct individuals, depending on the environmental context, genetic predisposition, and critical time window of neurodevelopment (Cicchetti & Rogosch, 1996;Marín, 2016). Besides this variability, the overwhelming majority of neuroimaging studies still focus on average group analysis and discard the individual differences (Marquand et al., 2019). To circumvent this constraint, the normative modeling methods offer individual-specific statistical inferences based on a previously learned pattern of typicality (Marquand et al., 2019). One notable aspect of these methods is their capacity to identify and delineate individual atypicality without relying on a uniform neurobiological pattern among all subjects (Marquand et al., 2019).

Recently, neuroimaging studies have used normative modeling to detect and segment brain lesions based on deep generative models (Baur et al., 2021;Chen et al., 2020;Pinaya et al., 2022). These algorithms use data from only typically developing (TD) subjects to learn a probability density function that reflects the scenario of typicality (Pinaya et al., 2022). When assessed by a model, the atypical subjects (with pathological features) are detected as deviations from typicality. As these models use unsupervised methods, they have the advantage of not depending on labeled or anomaly examples in the training phase (Pinaya et al., 2022). Furthermore, all the atypical neuroanatomy variability is analyzed at the individual level. That is, the heterogeneity of the individual differences is captured, allowing the mapping of different neurological conditions.

In this context, a recent study byPinaya et al. (2022)used an innovative approach to achieve state-of-the-art performance for detecting and segmenting brain lesions and tumors from T2-Flair MRI. Shortly after, this approach was adapted to detect early schizophrenia from brain T1 structural MRI (sMRI) (Da Costa et al., 2022), exceeding the performance of previous methods. The architecture used in both studies is composed of two models. The first, called Vector Quantized Variational Autoencoder (VQ-VAE) (Razavi et al., 2019;Van Den Oord et al., 2017), is responsible for reducing the size of the input MRI from millions of voxels to a representation of thousands of latent discrete codes. The second, a Generative Pre-trained Transformer (GPT) (also known as autoregressive Transformer) (Radford et al., 2018,^2019^;Vaswani et al., 2017) uses the encoded representation generated by the VQ-VAE to learn a probability density function of the typical brain. The VQ-VAE skill to reduce dimensions and tokenize images and the GPTs’ ability to map input data relationships regardless of distance makes them optimal candidates for neuroimaging tasks (Graham et al., 2022;Pinaya et al., 2022).

The results achieved by the GPT-based normative models to detect and segment brain anomalies (Pinaya et al., 2022) and to detect early schizophrenia (Da Costa et al., 2022) made us question whether this approach could be effective for the investigation of dimensional psychopathologies. Datasets such as the Adolescent Brain Cognitive Development (ABCD) (Casey et al., 2018) and the Brazilian High-Risk Cohort Study (BHRCS) (Salum et al., 2014) include T1 sMRI and scores of psychiatric symptoms from youths. These symptoms were measured by the Child Behavior Checklist (CBCL), which is composed of a list of questions answered by the parents (or caregivers) of youths (Achenbach & Rescorla, 2001). Each answer is summed to a score of the symptom categories: aggressive behavior, anxious/depressed, attention problems, rule-breaking behavior, somatic complaints, social problems, thought problems, and withdrawn (Achenbach & Rescorla, 2001). On a broader level, these categories are combined in scales of internalizing and externalizing problems, and all problems are totalized as the CBCL total score (Achenbach & Rescorla, 2001). Besides assessing these behavioral symptoms, we also investigated how the trained models performed evaluating scores of Attention Deficit Hyperactivity Disorder (ADHD) and diagnosis of Autism Spectrum Disorder (ASD). Whereas the behavioral symptoms captured by the CBCL and ADHD scores may capture mild or early-stage conditions, the positive diagnosis of ASD can capture an already established disorder. BHRCS, ABCD, Attention Deficit Hyperactivity Disorder (ADHD-200), and Autism Brain Imaging Data Exchange II (ABIDE-II) consortiums provide datasets for these investigations (Di Martino et al., 2017;Milham et al., 2012).

This study investigates if a normative architecture composed of the VQ-VAE and GPT models is able to detect psychiatric symptoms or disorders from brain sMRI of youths. We assess each brain Region of Interest (ROI), from the studied datasets, for associations with the CBCL groups of symptoms, ADHD scores, and diagnostics of ASD.

## 2 Materials and Methods

**Fig. 1.**
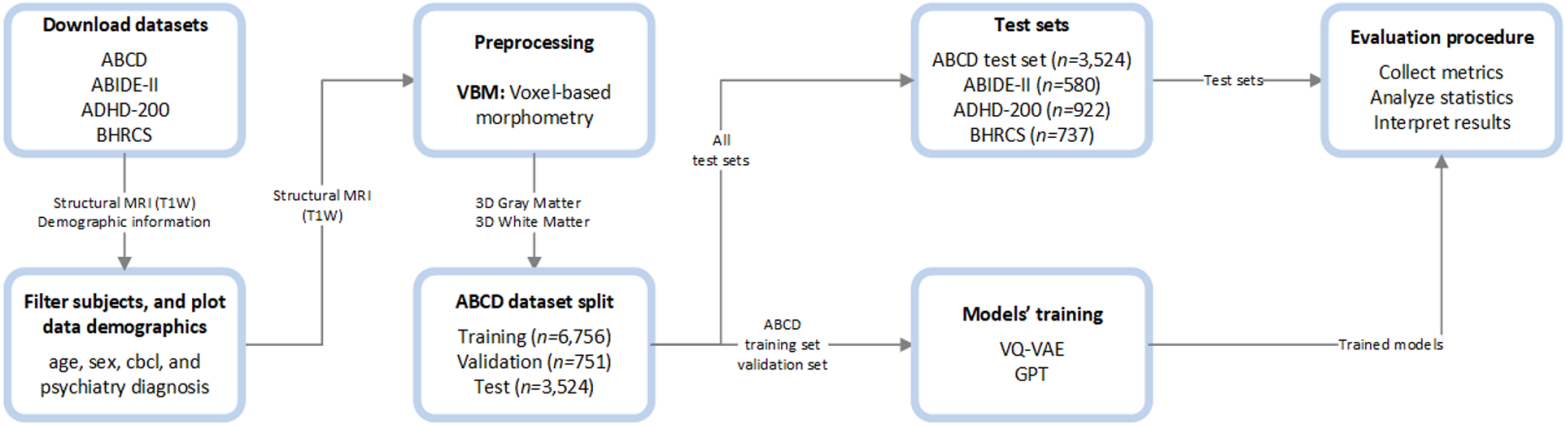
Processing pipeline. First, the datasets were downloaded, filtered, and preprocessed using Voxel-based morphometry. Then, the ABCD dataset was split into test and training/validation sets, whereas out-of-sample datasets were fully reserved for the test phase. After the models’ training, the results were collected, analyzed, and interpreted from the test sets only.

The adopted methods followed a logical sequence of steps. First, the data were downloaded, filtered, preprocessed, and split into training, validation, and test sets. Then, models were configured and trained from the training and validation sets. Finally, the trained models evaluated the unexplored test sets, producing metrics to interpret the results.Figure 1presents an overview of the processing steps.

### 2.1 Data description

The studied data include two datasets containing scores and diagnoses of psychiatric disorders, respectively: Attention Deficit Hyperactivity Disorder (ADHD-200) and Autism Brain Imaging Data Exchange II (ABIDE-II), and two neurodevelopmental studies containing measures of dimensional psychopathology: the Brazilian High-Risk Cohort Study (BHRCS) and Adolescent Brain Cognitive Development (ABCD) (Casey et al., 2018;Di Martino et al., 2017;Milham et al., 2012;Salum et al., 2014). SeeSupplemental Information(section 5.1) for further details. The data were collected and made publicly available according to the guidelines, and approval was provided by the local ethics committee for each project.

### 2.2 Participants

Our study targeted neurodevelopmental processes in youth. Thus, we selected subjects younger than 20 years of age and used only the last collected sMRI image of each subject who participated in the baseline scanning session. A demographic overview of the data is shown inFigure 2andTable 1. SeeSupplemental Information(section 5.2) for further details.

**Fig. 2.**
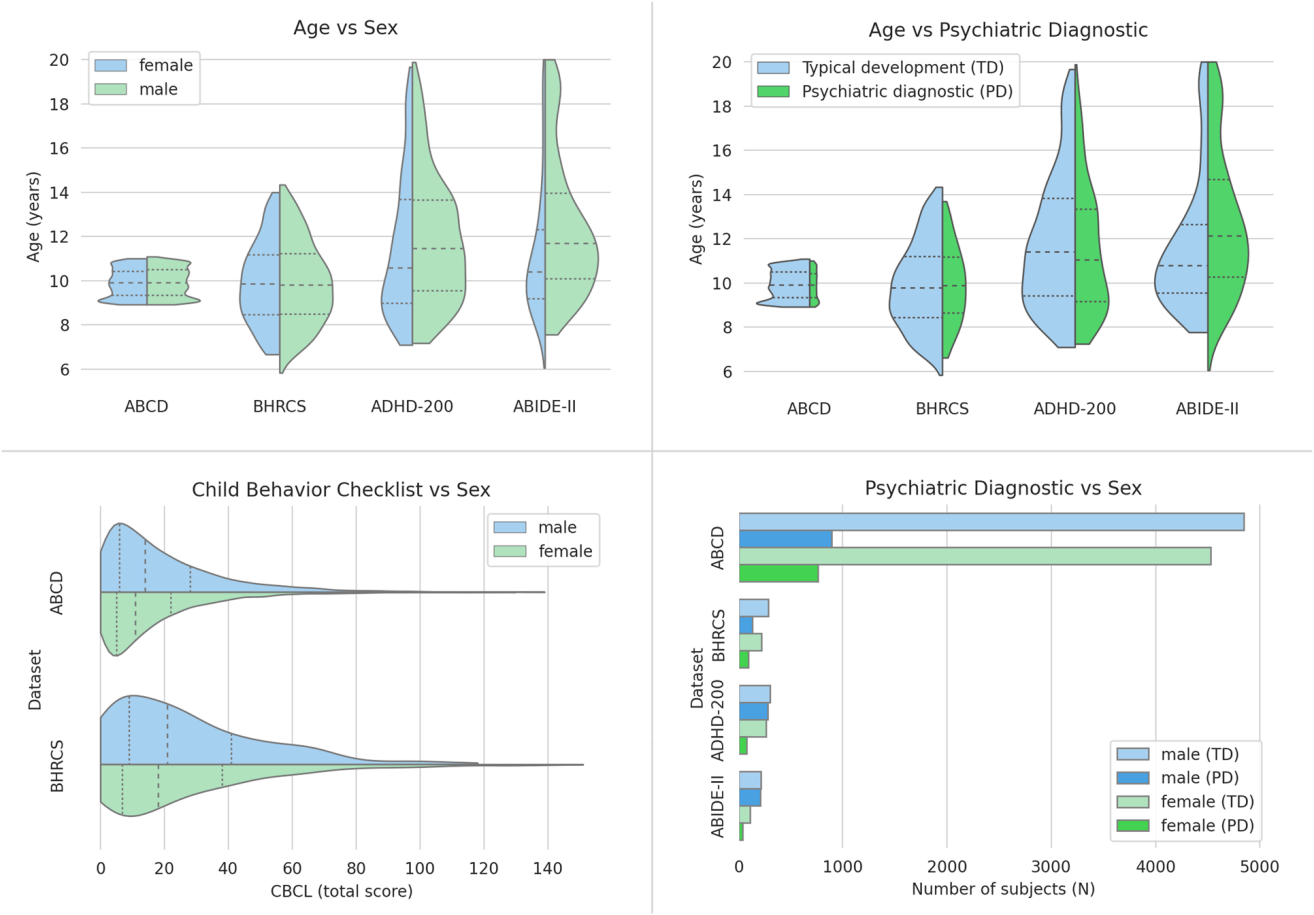
Datasets demographic distribution. Note the different distributions of age, sex, and psychiatric diagnostics among the datasets. The PD acronym represents different psychiatric diagnostics in each dataset (i.e., ASD for ABIDE-II, ADHD for ADHD-200, and all the DSM-IV or DSM-V diagnostics, respectively, for BHRCS and ABCD). Note that the distributions of CBCL scores present lower values in ABCD than in BHRCS. This is because the BHRCS screening protocol prioritized participants with higher risks of developing psychiatric disorders. Dotted lines mark the quartiles. Acronyms: CBCL = Child Behavior Checklist, TD = Typically Developing, PD = Psychiatric Diagnostic.

**Table 1.** Demographic information.

Data set	N	Age, y ± SD	Male, %	PD, %	CBCL ± SD
ABCD	11,031	9.9 ± 0.6	52.0%	15.0%	18.1 ± 17.9
ABIDE-II	580	12.1 ± 3.2	73.8%	43.3%	-
ADHD-200	922	11.7 ± 3.0	63.1%	38.7%	-
BHRCS	737	9.9 ± 1.9	57.1%	30.5%	27.1 ± 25.2

The sample size is denoted by N. Age is presented in years ± standard deviations. The CBCL total score is on a raw scale ± standard deviations. Subjects with any psychiatric disorder are grouped in PD. For ADHD-200, PD contains the subtypes of ADHD, and for ABIDE-II PD includes different levels of the autism spectrum. For ABCD and BHRCS, PD contains subjects with at least one mental disorder according to DSM-V (ABCD) and DSM-IV (BHRCS). Note the datasets differences for sample size, age range, psychiatric diagnostic, and CBCL. The CBCL scores present higher values in BHRCA than in ABCD. This is because the BHRCS screening protocol prioritized participants with higher risks of developing psychiatric disorders. Acronyms: CBCL = Child Behavior Checklist, PD = Psychiatric Diagnostic.

### 2.3 MRI preprocessing

The collected sMRI were preprocessed using the Voxel-Based Morphometry (VBM) (Ashburner & Friston, 2000). In brief, the VBM spatially normalizes MRI images to the same stereotactic space, allowing the extraction of different brain tissues from images partitioned with correction for nonuniform intensity variations (Ashburner & Friston, 2000). SeeSupplemental Information(section 5.3) for further details.

### 2.4 Model architecture and training

This study follows and adapts a network architecture recently proposed byPinaya et al. (2022)(open access) to detect pathological lesions from brain images. Their approach became state-of-the-art in brain anomaly detection using CT and FLAIR imaging data. In brief, the architecture is composed of two models, a VQ-VAE (Pinaya et al., 2022;Razavi et al., 2019;Van Den Oord et al., 2017) and a GPT (also known as an autoregressive Transformer) (Pinaya et al., 2022;Radford et al., 2019;Vaswani et al., 2017). The VQ-VAE learns a latent discrete representation of the brain, while the GPT models the likelihood of occurrence of each discrete element. Like in the referenced work (Pinaya et al., 2022), our approach is normative as we use only typical subjects (i.e., normal) to train the models. In the test phase, both atypical and typical subjects are evaluated. As the trained models learn exclusively from the typical, the atypicality is detected as a deviation from the learned pattern of typicality. Therefore, it is expected that during the test phase, the atypical brain regions present typicality scores that differ from the ones of typical patterns. Similar to other normative models, our approach has the advantage of identifying and outlining individual atypicality without relying on a uniform neurobiological pattern among all subjects (Marquand et al., 2019). Moreover, the atypical neuroanatomy variability is analyzed at the individual level, and the heterogeneity of the individual differences is captured, allowing the mapping of different neurological conditions. The architecture is depicted inFigure 3. SeeSupplemental Information(section 5.4) for further details.

**Fig. 3.**
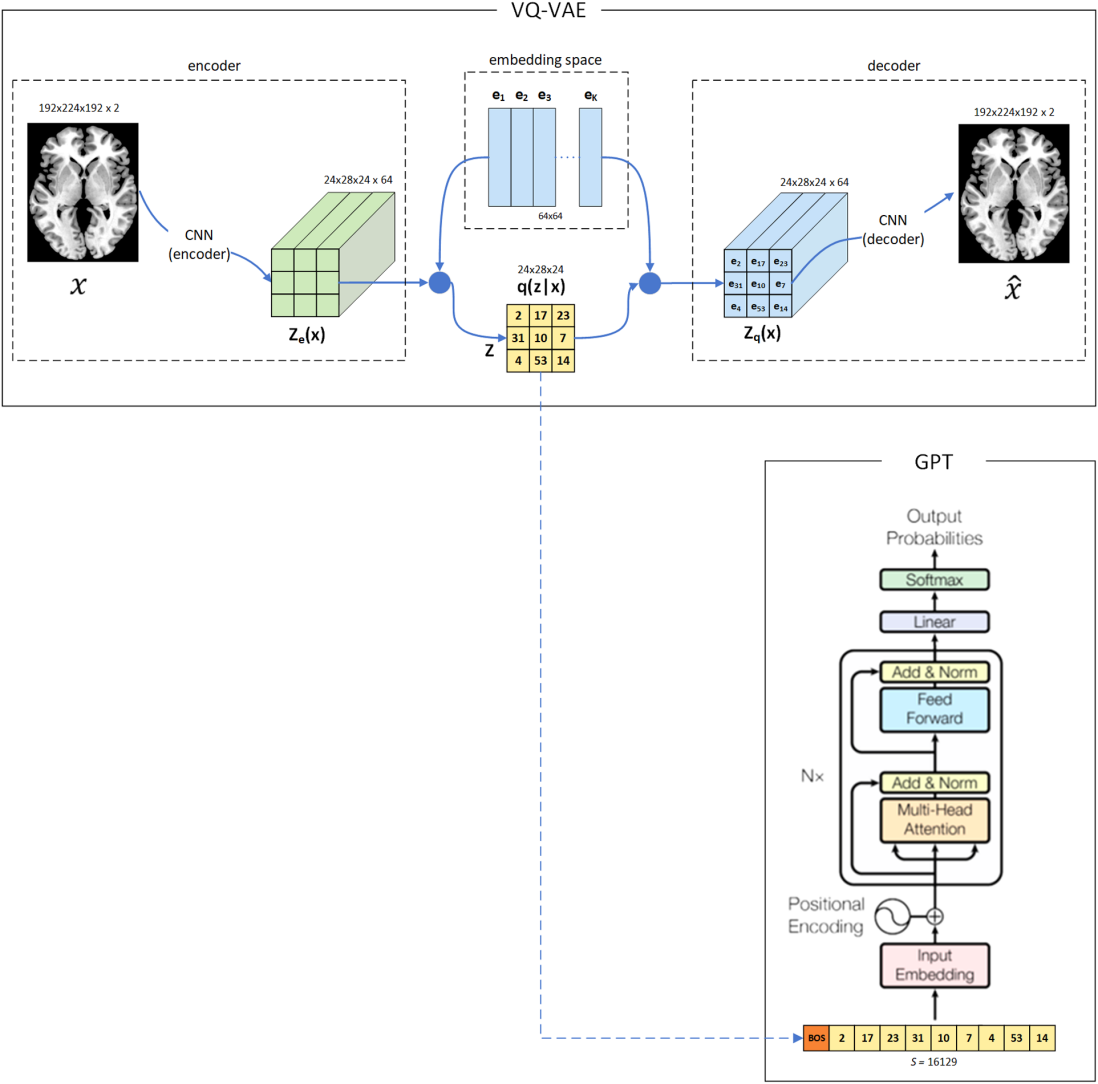
Network Architecture. The VQ-VAE encoder uses brain imagesxto map the observations to a latent representation${\text{z}}_{\text{e}}(\text{x})$of the brain. Then, the embedding space is used as a codebook by the VQ-VAE to transform${\text{z}}_{\text{e}}(\text{x})$in discrete representations ofq(z| x). This is done by selecting the${\text{e}}_{\text{x}}$vectors that are more similar (closer) to each${\text{z}}_{\text{e}}(\text{x})$element. This encoding process reduces the input dimension ofxfrom 14.2 million voxels to 16.1 thousand latent discrete codes inq(z| x). Next, theq(z| x)codes are serialized to train a GPT that outputs the probabilities (likelihoods) of the discrete elements. Therefore, the likelihood of each discrete element outputted by the GPT corresponds to a specific brain segment of the input observation. The decoder part of the VQ-VAE that reconstructs the codesq(z| x)in a reconstructed image$\hat{\text{x}}$is necessary for the learning (optimization) process of the VQ-VAE. For didactic purposes, the scheme depicts an architecture for 2 d input images. The accurate dimensions of the architecture are shown right up the illustrations ofx,${\text{z}}_{\text{e}}(\text{x})$,q (z| x),${\text{z}}_{\text{q}}(\text{x})$and$\hat{\text{x}}$. This figure was adapted from the originals (Van Den Oord et al., 2017;Vaswani et al., 2017).

### 2.5 Evaluation procedure

The trained network predicts the likelihood of typicality of the downsized and quantized brain segments, where each VQ-VAE quantized brain segment (from the dimension of 24x28x24) corresponds to a specific serialized GPT vocabulary token (in the sentence of 16,128 tokens) (seeFig. 3). Then, the likelihoods of these brain segments (i.e., vocabulary tokens) are grouped and averaged within the regions of the AAL3 3D brain atlas (Rolls et al., 2020) to provide ROI identification and allow literature comparability. The metrics are extracted from unbiased and unexplored data, according to the plan presented inTable 2. SeeSupplemental Information(section 5.5) for further details.

**Table 2.** Evaluation plan.

Dataset	Target	Target type	Metrics	α
ABCD test set	CBCL symptom groups	Numeric	r, p-value	<0.05
BHRCS	CBCL symptom groups	Numeric	r, p-value	<0.05
ADHD-200	ADHD symptom groups	Numeric	r, p-value	<0.05
ABIDE-II	ASD diagnosis	Binary	AUC, p-value	<0.05
All above	Chronological age	Numeric	r, p-value	<0.05

CBCL symptom groups include: aggression, anxiety, depression, rule break, somatic, attention, social, thought, opposite, conduct, others, internalizing, externalizing, and total scores. ADHD symptom groups include: inattention, hyperactivity/impulsivity, and ADHD-index scores. p-Values are Bonferroni-corrected before checking the accepted statistical significance level (α: alpha). The AUC p-value is calculated using permutation tests (Combrisson & Jerbi, 2015) (with 1,000 permutations). Acronyms: r = Pearson´s correlation, AUC = area under the receiver operating characteristic curve.

### 2.6 Models’ interpretability

One of the benefits of the adopted approach is that the GPTs output likelihoods of typicality that were indirectly obtained from brain segments. That is, the brain sMRI input is downscaled to latent discrete codes that are serialized to train GPT models. Therefore, each likelihood of the vector outputted by the GPT, during prediction, represents a specific brain segment. Thereby, we can reshape and upscale the GPTs’ outputs to obtain the map of likelihoods of typicality (per voxel) in the original 3 d input space of the brain sMRI. SeeSupplemental Information(section 5.6) for further details.

### 2.7 Software and hardware specification

All source codes are publicly available (https://github.com/SergioLeonardoMendes/normative_psychiatry). SeeSupplemental Information(section 5.7) for further details.

## 3 Results

After successfully executing the data preprocessing and model training steps, the evaluation and interpretation procedures were conducted as planned (seesection 2).

During the evaluation of the ABCD test set (*n = *3,524), the whole-brain predicted likelihood of typicality presented a statistically significant correlation with CBCL´s social problems scale (corrected p-value = 0.006). When assessing brains’ parcellations, significant correlations were found for the CBCL symptom groups of: total, externalizing, rule-breaking, aggressive, conduct, and social problems subscales. The detected associations between brain ROIs and psychiatric symptoms are presented inTable 3andFigure 4a. See supplementaryTable S1for statistical metrics.

**Fig. 4.**
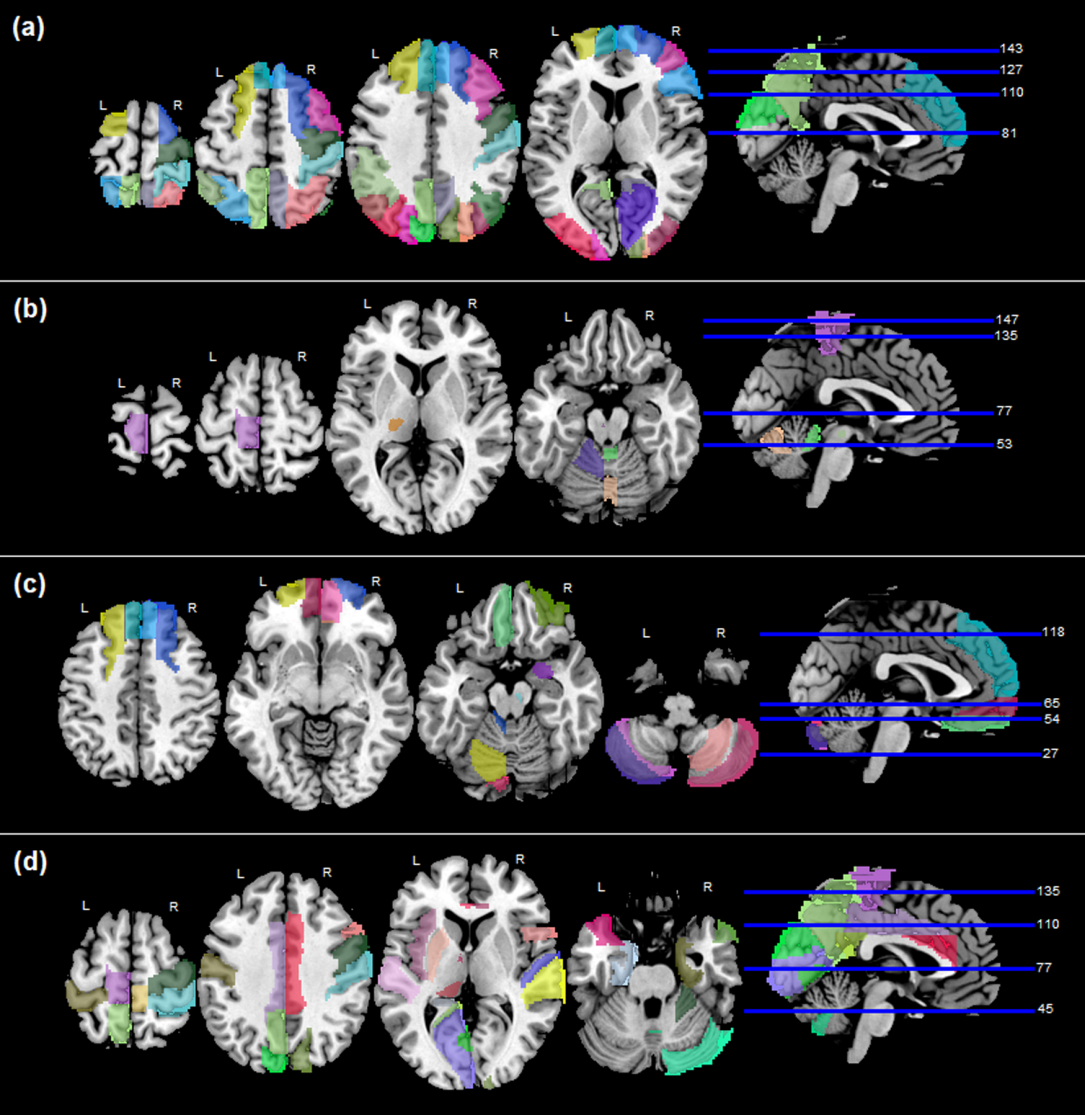
Brain regions associated with psychiatric symptoms or disorders: (a) CBCL symptoms in ABCD test set, (b) CBCL symptoms in BHRCS dataset, (c) ADHD symptoms in ADHD-200 dataset, (d) ASD diagnostic in ABIDE-II. The mapped regions are statistically significant after correcting the p-values by Bonferroni’s method.

**Table 3.** Brain regions versus CBCL symptom groups in the ABCD test set.

Brain region	Social	Conduct	Aggressive	Rule break	Externaliz.	Total
Whole brain	0.060					
Precentral gyrus (right)	0.074					
Sup. frontal gyrus, dorsolat. (left)	0.084	0.073		0.076	0.075	0.075
Sup. frontal gyrus, dorsolat. (right)	0.080					
Sup. frontal gyrus, medial (left)	0.074	0.078		0.080	0.075	
Sup. frontal gyrus, medial (right)	0.081					
Middle frontal gyrus (right)				0.078		
Inf. frontal gyrus, triang. part (right)				0.074		
Calcarine fissure surr. cortex (right)	0.076					
Cuneus (left)	0.081					
Cuneus (right)	0.098					
Superior occipital gyrus (left)	0.091					0.075
Superior occipital gyrus (right)	0.101					
Middle occipital gyrus (left)	0.073					
Middle occipital gyrus (right)	0.081					
Postcentral gyrus (right)	0.085					
Superior parietal gyrus (left)	0.123	0.080	0.082	0.087	0.089	0.098
Superior parietal gyrus (right)	0.102			0.080	0.079	0.081
Inferior parietal gyrus (left)	0.090					
Angular gyrus (left)	0.077					
Angular gyrus (right)	0.085					
Precuneus (left)	0.096			0.082		
Precuneus (right)	0.095					0.077

Numbers indicate the statistically significant Pearson’s correlation between regions of interest and groups of CBCL symptoms, after correcting p-values by Bonferroni’s method. Note that all brain areas associated with the CBCL groups of symptoms are cortical regions. Some brain regions are correlated with more than one group of symptoms, including the groups of externalizing and total scores. The superior parietal gyrus (left) and superior frontal gyrus (dorsolateral left) are the regions that correlated to the highest number of symptom groups.

For the BHRCS dataset (n = 737), the predicted likelihood of typicality of whole brains has not presented significant correlations to psychiatric symptoms after statistical corrections. When assessing the symptoms per brain region, significant correlations were found for the CBCL symptom groups of: social, thought, somatic, depression, anxiety, internalizing, and total scores. The brain ROIs and their association to psychiatric symptoms are presented inTable 4andFigure 4b. See supplementaryTable S2for statistical metrics.

**Table 4.** Brain regions versus CBCL symptom groups in the BHRCS dataset.

Brain region	Social	Thought	Somatic	Depression	Anxiety	Internaliz.	Total
Cerebellar lobules IV and V (left)			0.158				
Cerebellar vermis lobule III	-0.188			-0.160	-0.160	-0.182	-0.173
Cerebellar vermis lobule VI			0.171				
Thalamus ventral posterolat (left)	0.163	0.179	0.169			0.171	0.180
Ventral tegmental area (left)						0.164	
Red nucleus (left)			0.161			0.169	0.162

Numbers indicate the statistically significant Pearson’s correlation between regions of interest and groups of CBCL symptoms, after correcting p-values by Bonferroni’s method. Note that all brain areas associated with the CBCL groups of symptoms are subcortical regions. Some brain regions are correlated with more than one symptom group, including the internalizing and total scores groups. The cerebellar vermis (lobule III) and thalamus ventral posterolateral (left) are the regions that correlated to the highest number of symptom groups.

Evaluating the ADHD-200 dataset (n = 922), the whole-brain predicted likelihood was not found to be correlated to the scores of inattentive, hyper-impulsive, or ADHD index. However, significant correlations were found when assessing the ADHD score groups by brain ROIs. The brain regions and their detected associations are presented inTable 5andFigure 4c. SeeTable S3for statistical metrics.

**Table 5.** Brain regions versus ADHD scores in the ADHD-200 dataset.

Brain region	Hyperactive / impulsive	Inattentive	ADHD index
Superior frontal gyrus, dorsolateral (left)	-0.203	-0.182	
Superior frontal gyrus, dorsolateral (right)	-0.266	-0.226	
Superior frontal gyrus, medial (left)	-0.229	-0.192	
Superior frontal gyrus, medial (right)	-0.266	-0.231	
Superior frontal gyrus, medial orbital (left)	-0.178		
Superior frontal gyrus, medial orbital (right)	-0.200		
Gyrus rectus (left)	-0.185		
Anterior orbital gyrus (right)	-0.204	-0.176	
Amygdala (right)	-0.170		
Cerebellar crus I (left)	-0.177		
Cerebellar crus II (left)	-0.263	-0.235	
Cerebellar crus II (right)	-0.187		
Cerebellar lobule III (left)	-0.174		
Cerebellar lobule VI (left)	-0.177		
Cerebellar lobule VII (left)	-0.248	-0.219	
Cerebellar lobule VIII (right)	-0.191		
Thalamus ventral anterior (right)	0.181		
Thalamus pulvinar lateral (left)	0.190		
Anterior cingulate cortex, pregenual (right)	-0.182		
Anterior cingulate cortex, supracallosal (right)	-0.176	-0.171	
Substantia nigra, pars compacta (right)	-0.248	-0.228	-0.170

Numbers indicate the statistically significant Pearson’s correlation between regions of interest and groups of CBCL symptoms, after correcting p-values by Bonferroni’s method. Note that the brain areas associated with ADHD scores are part of the cortical and subcortical regions. Some regions are correlated with both inattentive and hyperactive/impulsive scores. The substantia nigra pars compacta (right) is the only region correlated with the ADHD index score.

We also used the model’s likelihood of typicality to assess ASD diagnoses. From the ABIDE-II dataset (*n*= 580), the whole-brain predicted likelihood of typicality was found to be discriminant of ASD (AUC = 0.60, p-value <0.001). The evaluation of significant AUC by brain regions also found several ROIs as being correlated with ASD diagnosis. These ROIs are shown inTable 6andFigure 4d. SeeTable S4for the AUC metrics of ASD diagnosis.

**Table 6.** Brain regions correlated with ASD in the ABIDE-II dataset.

Brain region	ASD diagnosis
Whole brain	0.600
Precentral gyrus (right)	0.594
Inferior frontal gyrus, opercular (right)	0.582
Cingulate gyrus, middle (left / right)	0.614 / 0.584
Cingulate gyrus, posterior (left)	0.572
Insula (left)	0.578
Hippocampus (left)	0.597
Parahippocampal gyrus (left / right)	0.627 / 0.576
Calcarine fissure and surrounding cortex (left)	0.606
Cuneus (left / right)	0.580 / 0.575
Precuneus (left)	0.627
Lingual gyrus (left)	0.588
Postcentral gyrus (left / right)	0.599 / 0.626
Paracentral lobule (left / right)	0.582 / 0.604
Putamen (left)	0.591
Heschl’s gyrus (right)	0.620
Temporal gyrus, superior (left / right)	0.616 / 0.609
Temporal pole (left / right)	0.605 / 0.594
Cerebellar crus I (right)	0.585
Cerebellar lobules IV/V and IX (right)	0.606
Cerebellar vermis (lobule VIII)	0.599
Thalamus anterior, ventral (left)	0.586
Thalamus pulvinar, medial (left)	0.603
Anterior cingulate cortex, supracallosal (left / right)	0.587 / 0.593
Ventral tegmental area (right)	0.591
Substantia nigra, pars compacta (right)	0.569

Numbers indicate the statistically significant AUC between brain ROIs and ASD diagnosis, after correcting p-values by Bonferroni’s method.

The likelihood of typicality was also used to assess the brain aging as a potential confounder to the other measurements. This analysis showed the whole brain as correlated with aging for all but the ABCD dataset. Some brain ROIs were also correlated with brain aging as shown inTable 7. See supplementaryTable S5for the brain aging statistical metrics.

**Table 7.** Brain regions associated to chronological aging in each dataset.

Brain region	ABCD	BHRCS	ADHD-200	ABIDE-II
Whole brain		-0.07	-0.19	-0.33
Thalamus ventral lateral (right)	0.075			0.261
Thalamus ventral anterior (right)	0.104			0.279
Red nucleus (right)	0.076			
Globus pallidus (right)		-0.195		
Cerebellar lobules IV / V (right)		-0.184		-0.300
Cerebellar lobule VI (right)		-0.171		-0.257
Cerebellar vermis (lobules I and II)		-0.211		-0.218
Thalamus intralaminar (left)		-0.194		-0.194
Thalamus reuniens (left)		-0.171		
Ventral tegmental area (right)		-0.163		-0.357
Thalamus pulvinar anterior (left)			-0.170	-0.270
Precentral gyrus (left / right)				-0.194 / -0.270
Frontal gyrus superior (right)				-0.238
Frontal gyrus middle (right)				-0.150
Frontal gyrus inferior opercular (right)				-0.224
Rolandic operculum (left / right)				-0.217 / -0.234
Supplementary motor area (left / right)				-0.185 / 0.212
Superior frontal gyrus (left)				-0.171
Orbital gyrus, anterior (left / right)				-0.243 / -0.309
Orbital gyrus, lateral (left / right)				-0.199 / -0166
Hippocampus (left)				-0.226
Cuneus (left / right)				-0.158 / -0.151
Angular gyrus (left)				0.213
Precuneus (left)				-0.177
Paracentral lobule (left / right)				-0.245 / -0.153
Caudate nucleus (left / right)				-0.293 / -0.206
Globus pallidus (left)				0.225
Heschl’s gyrus (right)				-0.212
Temporal pole (superior, middle - left and right)				-0.186 ~ -0.337
Cerebellar lobules (all - left and right)				-0.156 ~ -0.369
Cerebellar vermis (III, VIII, IX, X)				-0.186 ~ -0.364
Thalamus (anterov., reuniens, lgn, pulvinar, left and right)				-0.166 ~ -0.352
Substantia nigra (p. compacta, p. reticulate, left and right)				-0.213 ~ -0.337
Locus coeruleus (left and right)				-0.200

Numbers indicate statistically significant Pearson’s correlations between regions of interest and chronological aging, after correcting p-values by Bonferroni’s method.

## 4 Discussion

This study investigated whether a normative architecture composed of the VQ-VAE and GPT models could predict typicality scores that are correlated with psychiatric symptoms and disorders, from brain sMRI of youths. Models were trained from typical development subjects (only), using the lower quartiles of CBCL total score from the ABCD dataset. Next, the trained models predicted the likelihoods (of typicality) for the whole brain and each brain segment of the tested subjects. During the evaluation, the likelihoods of typicality were assessed for associations with (i) the scores of CBCL symptom groups (for the ABCD test set and BHRCS dataset), (ii) the scores of ADHD (for ADHD-200 dataset), (iii) the diagnostics of ASD (for the ABIDE-II dataset), and (iv) the chronological age (for all datasets). The resulting p-values were corrected by Bonferroni’s method, and the statistically significant associations were identified and charted.

This approach identified that the whole brain’s likelihood of typicality was correlated with social problems (for the ABCD test set), ASD diagnosis (for the ABIDE-II dataset), and age (for the BHRCS, ADHD-200, and ABIDE-II datasets). The analysis by brain region linked different brain ROIs to several CBCL scales, ADHD scores, and ASD diagnostic.

To the best of our knowledge, there are no successful studies assessing all dimensional groups of CBCL symptoms, from all brain regions, based exclusively on sMRI. A recent study tried to estimate the CBCL total score from the ABCD and BHRCS datasets using structural MRI; however, the authors’ attempt was unsuccessful (Mendes et al., 2023). The same applies to ADHD, where most MRI studies explore approaches based on classification (instead of dimensional score estimation). Therefore, this study will discuss not only based on structural MRI studies, instead, sometimes we will need to resort to the classical neuroanatomy literature as well as other modalities of MRI (e.g., resting state and functional MRI).

The assessment of the CBCL symptom groups for ABCD exhibited ROIs that correlated with problems of socializing, conduct, aggressiveness, rule-breaking, externalizing, and total symptoms. In contrast, the BHRCS subjects presented ROIs that correlated with problems of socializing, thought, somatic, depression, anxiety, internalizing, and total symptoms (seeTables 3and4). Interestingly, all ROIs that emerged from the ABCD are cortical, whereas all ROIs shown for the BHRCS are subcortical regions. While all ABCD symptom groups are externalizing, the BHRCS symptom groups are above all internalizing. In other words, the ABCD exhibited cortical regions correlated with externalizing symptoms, whereas the BHRCS presented subcortical regions mostly correlated with internalizing symptoms.

Classical neuroanatomy literature indicates that the ROIs highlighted in ABCD, which are associated with more than one group of symptoms (seeTable 3), process mainly multimodal associative information (Martin, 2014). The superior and inferior (angular) parietal gyri are sites of the superior order somatosensory posterior-parietal associative cortex, and parieto-temporo-occipital associative cortex, respectively (Martin, 2014). These areas integrate functions for the visuomotor spatial consciousness, perception, vision, reading, and speech (Martin, 2014). The superior, medial, and inferior frontal gyri host the frontal eye fields and the prefrontal associative cortices, which are responsible for thinking, cognition, and behavioral and movement planning (Martin, 2014). Together, these associative regions are also part of the oculomotor and associative brain loops (Martin, 2014). While the oculomotor loop searches and finds relevant information in a scene through the saccadic eye movements, the associative loop works for the cognition and executive functional behaviors as well as in planning behavioral strategies (Martin, 2014).

Other regions highlighted in ABCD, such as the precentral, postcentral, precuneus, cuneus, occipital gyri, and calcarine fissure cortex, which are primary and unimodal association cortices (Kandel et al., 2000), also participate in the information processing. That is, the primary sensory cortices (e.g., postcentral gyrus and calcarine fissure cortex) send information (e.g., somatosensory and visual) to unimodal association cortices (e.g., superior parietal and unimodal occipital gyri) to finally arrive at the multimodal associative cortices (e.g., parieto-temporo-occipital and prefrontal associative cortices) (Kandel et al., 2000). After processing sensory information, the multimodal associative cortices can transmit information to unimodal cortices and finally send instructions to the primary motor cortex (i.e., precentral gyrus) to produce body movements (Kandel et al., 2000). The ROIs highlighted in ABCD appear to be coherent with regions expected to participate in the information processing of the related psychiatric symptoms (i.e., social, conduct, aggressive, rule break, and externalizing).

In the BHRCS dataset, the associations came exclusively from subcortical structures, including the cerebellar lobules, thalamus, red nucleus, and ventral tegmental area (seeTable 4). Despite the well-recognized role of the cerebellum in sensorimotor functions, studies also indicate that it plays an important role as a modulator of emotional processing, producing both excitatory and inhibitory tones via its connections to the ventral tegmental area (Sacchetti et al., 2009;Shakiba, 2014). Among other neuropsychiatric symptoms, cerebellar lesions were found to elicit problems of depression, anxiety, socializing skills, and somatic manifestations, therefore, consistent with our findings (Schmahmann et al., 2007;Shakiba, 2014). In addition, the thalamus ventral posterolateral has a relay role, projecting peripheral information (e.g., tact, members position, and temperature sensation) to the somatosensory cortex (Martin, 2014). Interestingly, our study found this ROI is associated with somatic, thought, and social symptoms. Analyzing the somatic group of symptoms, the highlighted regions (i.e., cerebellum, thalamus lateral, and red nucleus) were previously found to be associated with somatic pain processing in functional MRI studies (Bingel et al., 2003;Dunckley et al., 2005). The role of the thalamus lateral is suggested as relaying spatial information of selective nociceptive stimuli to the somatosensory cortex to provide pain localization (Bingel et al., 2003). Overall, the ROI associations found in BHRCS are consistent with the related literature.

A major issue was that the findings of BHRCS did not replicate the ones of ABCD. We conjecture that this occurred due to the differences in the demographic data distribution of these datasets, especially the age ranges (seeFig. 2). Neural development involves highly coordinated and sequenced events of both progressive (myelination) and regressive (synaptic pruning) processes (Silk & Wood, 2011). These brain transformations affect GM and WM densities at different rates, in a regionally and temporally specific way (Gogtay et al., 2004;Silk & Wood, 2011). Therefore, the different age ranges of the ABCD and BHRCS may have influenced the non-replicability of the results in these datasets. Another limitation is due to the multiple distinct scanner models used to collect the sMRI in each dataset (seeSupplemental Information, section 5.1). A recent study indicates that differences in the acquisition parameters of scanners represent a major limitation for the generalizability of brain models (Jirsaraie et al., 2023). The set of scanner models used to train our artificial neural network (from ABCD dataset) is different from the set of scanners used by each of the testing datasets (i.e., BHRCS, ABIDE-II and ADHD-200). Therefore, the statistical effect size obtained from the testing datasets may have been reduced since our method does not control for the effect of scanners models. For instance, the scanners used to collect the ABCD data were 3T scanners whereas BHRCS employed 1.5T (exclusively). The higher magnetic field strength of the 3T models provides higher signal-to-noise and contrast-to-noise ratios (in comparison with 1.5T) (Duyn, 2012). This translates in less noise with increased image contrast and resolution (Duyn, 2012) for ABCD. That is, the 3T images (when compared to 1.5T) potentially capture more information on subtle contrast differences and small structural variations of the brain. Therefore, the differences in scanners’ acquisition parameters may also have influenced the non-replication of ABCD results on BHRCS data.

Analyzing the ADHD-200 dataset, the brain regions found to be associated with ADHD symptoms (seeTable 5) have already been reported as atypical in ADHD subjects (Krain & Castellanos, 2006;Posner et al., 2011,^2014^;Tomasi & Volkow, 2012;Zang et al., 2007). Studies with ADHD subjects show that the prefrontal cortex, basal ganglia, and cerebellum are known for presenting atypical volume when measured from structural MRI (Krain & Castellanos, 2006;Zang et al., 2007). In addition, the regions of the anterior cingulate cortex, and amygdala have been found to present abnormal activation in functional MRI studies of ADHD (Posner et al., 2011;Zang et al., 2007). Another functional MRI study found that the substantia nigra and its dopaminergic nigrostriatal pathways mature abnormally during childhood to adulthood in ADHD subjects (Tomasi & Volkow, 2012). Including the thalamus, most of these structures are part of the cortico-striato-cortical loops, which are neural circuits that are believed to show abnormal function in ADHD subjects (Posner et al., 2014). Taken together, the literature corroborates the regions highlighted in this study as being associated with ADHD.

When assessing ASD diagnosis from the ABIDE-II dataset, the whole-brain predicted likelihood was discriminant of both ASD (AUC = 0.60, p-value <0.001) and brain aging (r = -0.33, p-value <0.001) (see supplementaryTables S4-S5). Therefore, the discrimination of ASD through the whole brain may have been confounded by brain aging. The same applies to several brain parcellations that were correlated with both ASD and brain aging (see supplementaryTables S4-S5). However, some ROIs were exclusively discriminatory of ASD. These regions included the left insula, cingulate gyrus (left posterior left, middle left and right), parahippocampal gyrus (left and right), calcarine fissure and surrounding cortex (left), left lingual gyrus, postcentral gyrus (left and right), left putamen, and anterior cingulate cortex (supra callosal, left and right). Previous functional MRI studies found that these regions present lower activation in ASD patients when compared to TD subjects (Greene et al., 2011;Kana et al., 2007). More specifically, these regions are part of networks responsible for inhibitory control (Kana et al., 2007), and social orienting for spatial cueing (Greene et al., 2011). Therefore, the literature corroborates the ROIs found in our study as being discriminant of ASD.

The analysis of the correlation metrics (see supplementaryTables S1-S3) shows that the correlations’ direction changes when assessing different datasets. The correlations of the ABCD test set are positive (Table S1), whereas they are mixed (positive and negative) for BHRCS (Table S2), and almost entirely negative for the ADHD-200 dataset (Table S3). We conjecture that this occurs due to the differences in the data distribution (especially age) of these datasets (seeFig. 2). In other words, the assessment of subjects whose age is outside the ABCD age range may lead the correlations to be negative. As the typical neurodevelopment is coupled with aging (Gogtay et al., 2004;Silk & Wood, 2011), subjects presenting age deviations (from the training set) can have their normal neurodevelopment accounted as atypical by the models’ estimation. To investigate this scenario, we measured the association between the chronological age and the models’ likelihood of typicality (see supplementaryTable S5). The results indicated that differently of the ABCD test set (r = -0.01, p-value = 0.48), both the datasets BHRCS (r = -0.07, p-value = 0.046), ADHD-200 (r = -0.19, p-value < 0.001), and ABIDE-II (r = -0.33, p-value < 0.001) presented statistical significant association between age and the whole-brain likelihood of typicality. However, none of the brain ROIs associated with the CBCL groups of symptoms or ADHD (except the left thalamus pulvinar) showed a statistically significant association with age. We hypothesize that, although brain ROIs associated with psychiatric symptoms or ADHD are not significantly confounded by age, the influence of age across the brain may influence the direction of the correlations. Presumably, this scenario would not occur if the demographic data distribution of the training set and the evaluation datasets had the same shape.

Brain ROIs found to be correlated with chronological aging did not overlap across datasets. Although counterintuitive, this behavior was expected, since the studied datasets have distinct demographic distributions. Our model learned a typicality pattern from a training set with a specific distribution of age, sex, ethnicity, unknown comorbidities, and other demographic characteristics. During the evaluation, the trained model predicted typicality scores for datasets with data distributions distinct (i.e., out of the range) of that used for training. As previously mentioned, neural development involves highly coordinated and sequenced events that affect GM and WM densities at different rates, in a regionally and temporally specific manner (Gogtay et al., 2004;Silk & Wood, 2011). Therefore, given the unique characteristics of each dataset tested, it was expected that each would present its own set of ROIs correlated with chronological aging.

The approach adopted by this study presents several advantages over other methods. First, as the models’ learning is based only on TD subjects, the same trained model is capable of estimating different psychiatric conditions, from distinct datasets. This is because atypical subjects are detected as deviations from the learned pattern of typicality. Second, the models assess each region of the whole brain estimating its correspondent likelihood of typicality without bias to any previous hypothesis. Third, as psychiatric conditions are highly heterogeneous (Cannon & Keller, 2006;Cicchetti & Rogosch, 1996;Marín, 2016), our approach allows the detection and mapping of anomalies without requiring a consistent neurobiological signature among the evaluated subjects (Marquand et al., 2019). Fourth, the robustness of GPT (i.e., Transformer-based) normative models to map input data relationships regardless of their distance makes them great for neuroimaging tasks (Graham et al., 2022;Pinaya et al., 2021). This was evidenced in a recent study, where Transformer-based normative models outperformed other methods in the classification of early-stage schizophrenia from brain sMRI (Da Costa et al., 2022). Together, these characteristics made the proposed approach capable of estimating and mapping brain regions associated with psychiatric symptoms (i.e., CBCL symptom groups) from brain sMRI, for the first time.

Despite the advantages, some limitations need to be considered. The modest statistical effect sizes presented in the evaluation metrics (see results’ tables) make our strategy not feasible to classify (i.e., diagnose) subjects between typical and atypical (at least for the CBCL symptom groups, based on brain sMRI). Conversely, our approach is best suitable for mapping associations between brain ROIs and psychiatric conditions. Another constraint is related to the demographic distributions of data used for training and evaluation (seeFig. 2). Ideally, the shape of the distributions (e.g., age and sex) should be similar between training and evaluation data. Furthermore, this study did not analyze the subjects’ comorbidities as a potential confounder since this information is not available in the studied data. A possible way to circumvent the limitations related to differences in data distributions is to condition the models’ estimation based on context to have a demographic-dependent likelihood estimation (Da Costa et al., 2022). Presumably, the conditioning of the models by demographic information and other potential confounders (e.g., comorbidities and scanners’ model) should lead models to show more robust metrics (i.e., larger effect sizes). Another technique that promises to increase effect sizes is the likelihood ratio, which has the potential to emphasize in-distribution semantic components while demonstrating reduced sensitivity to high-frequency features shared across the population (Ren et al., 2019). Moreover, applying our approach to other modalities of data (e.g., functional MRI) is expected to produce better estimates due to the extra information (e.g., time-dependent brain activations) supplied to the models. Collectively, the study of neuroimaging from normative models based on GPT is a promising approach to investigate the gap between the phenotypes of psychiatric conditions and their neurobiological substrates.

## Supplementary Material

Supplementary Material

## Data Availability

The datasets used in this study were obtained from two public datasets: the Autism Brain Imaging Data Exchange II (ABIDE-II) and Attention Deficit Hyperactivity Disorder (ADHD-200); and from two datasets that required authorization: Adolescent Brain Cognitive Development (ABCD) and Brazilian High-Risk Cohort Study (BHRCS). ADHD-200 and ABIDE-II can be directly downloaded from the NeuroImaging Tools & Resource Collaboratory Image Repository (NITRC-IR:https://www.nitrc.org/ir/). For ABCD and BHRCS data sets, application and consortium approval of an NDA form are required. The data were collected and made publicly available according to the guidelines, and approval was provided by the local ethics committee for each project. Detailed information on these datasets and their acquisition parameters can be retrieved from ABIDE-II (http://fcon_1000.projects.nitrc.org/indi/abide/abide_II.html), ADHD-200 (http://fcon_1000.projects.nitrc.org/indi/adhd200/), ABCD (https://nda.nih.gov/abcd), and BHRCS (https://osf.io/ktz5h/wiki/home/). All source codes are publicly available (https://github.com/SergioLeonardoMendes/normative_psychiatry).
