# Supplementary material for: GPT-based normative models of brain sMRI correlate with dimensional psychopathology
Source: Imaging Neurosci (Camb). 2024 Jun 26;2:imag-2-00204. doi: 10.1162/imag_a_00204 (PMC12272264; doi:10.1162/imag_a_00204)
Supplement: Supplementary Material [file imag_a_00204-supp.pdf]

5. Supplementary Materials

5.1. Data Description

Only the first collection of data (i.e., baseline) of each dataset was used. The images were collected from different countries and locations, including eight sites for ADHD-200 and nineteen sites for ABIDE-II. Therefore, the scanners' acquisition parameters of ADHD-200 and ABIDE-II are varied, hosting head coils between 8 and 32 channels, from mostly 3T models. The data from ABCD were collected from 21 sites in the USA through multi-brand 3T scanners. The BHRCS data were obtained from two Brazilian cities from exclusively 1.5T scanners, comprising the 1.5T Signa HD and HDX models, from G.E. manufacturer. The acquisition parameters of the BHRCS are available in a study by Sato et al. (Sato et al., 2016). The acquisition parameters of ABCD can be retrieved at https://abcdstudy.org/images/Protocol_Imaging_Sequences.pdf. The scanners' parameters of ADHD-200 and ABIDE-II can be found at http://fcon_1000.projects.nitrc.org/indi/adhd200/ and http://fcon_1000.projects.nitrc.org/indi/abide/abide_II.html. The collection and availability of these data follow the guidelines and approval of each project's local ethics committee.

5.2. Participants

Subjects with no information on psychiatric evaluation (i.e., ADHD, ASD, CBCL, or TD), age, or sex, were discarded. The subjects of ABCD were split into three sets for training (*n*=6,756), validation (*n*=751), and testing (*n*=3,524) purposes. The CBCL total score was used as split criteria in the following manner. First, the examples were labeled as normal CBCL score (three lower CBCL quartiles) or high CBCL score (upper fourth CBCL quartile). Then, we assigned 10% of the normal and 100% of the high CBCL subjects to the test set. From the remaining 90% of normal CBCL subjects, one-tenth of the examples were assigned to validation and nine-tenths to the training set. The ABCD test set (*n*=3,524) and the complete datasets of ABIDE-II (*n*=580), ADHD-200 (*n*=922), and BHRCS (*n*=737) were reserved for the evaluation procedure (i.e., final tests). The purpose of reserving all high CBCL subjects for test sets is because the models are normative, trained exclusively with TD subjects. Therefore, during testing, high CBCL subjects are detected as deviations from the learned pattern of typicality.

5.3. MRI Preprocessing

The VBM preprocessing (Ashburner & Friston, 2000) was done through the Statistical Parametric Mapping software (SPM, version 12 release 7771) (Penny et al., 2007), available at https://www.fil.ion.ucl.ac.uk/spm/software/spm12/. The used parameters followed the SPM standard configuration pipeline (https://www.fil.ion.ucl.ac.uk/~john/misc/VBMclass15.pdf), according to the guide described by Mendes et al. (Mendes et al., 2021) (open access), but with voxels' size (resolution) set to 1 mm^3. Therefore, the preprocessed sMRI resulted in two matrices (i.e., GM and WM), each with a dimension of 181x217x181 voxels. We considered only the GM and WM to ensure that only brain tissue data would be delivered to the neural networks, eliminating the risk of biasing the models due to learning from non-brain tissues.

5.4. **Model Architecture and Training**

Differently of Pinaya et al. (Pinaya et al., 2022), we employed sMRI images preprocessed via VBM for training and evaluating the artificial neural networks. During the VQ-VAE learning process, the input data go from nearly 14.2 million voxels to 16.1 thousand latent discrete codes. The reduced dimension of this latent discrete representation allows the training of GPT that models the likelihood of occurrence of each discrete element.

As in the study of Pinaya et al. (Pinaya et al., 2022), we reordered the latent representations generated by the VQ-VAE to train multiple GPT models (based on different views of the input data). After training, we had nine GPT models each obtained from a specific view of the serialized latent representations. Then, the predictions from these models were averaged in an ensemble to evaluate the test samples. The number of training epochs was set to 50 for every trained model. As we employed a large training set (n=6756 examples), the models' learning curves showed that this number of epochs was empirically sufficient for the training phase (see supplemental figures S1 and S2).

5.5. **Evaluation procedure**

The typicality score of the whole brain is calculated from the sum of all tokens' likelihood logarithms. The use of logarithms is justified by its better numerical precision to represent the likelihood of each token that is very close to zero.

5.6. **Models' interpretability**

To allow interpretation and literature comparability, the brain maps (per voxel) are intersected with the AAL3 brain atlas (Rolls et al., 2020), and the likelihoods (per voxel) are averaged by each ROI of the brain atlas, producing per-ROI brain maps of likelihood. Then, we use the per-ROI likelihoods to measure the correlation with psychiatric scores, or the AUC of ASD diagnoses. Finally, the p-values (of correlations and AUCs) are Bonferroni-corrected and used to generate the above-chance maps of ROIs, i.e., maps of brain regions whose estimated likelihood of typicality may correlate with psychiatric scores or diagnostics.

5.7. **Software and hardware specification**

The sMRI preprocessing was done through the SPM12 v7771 software (https://www.fil.ion.ucl.ac.uk/spm/software/spm12/). All further steps used Python 3.8 and Pytorch 1.12.0 from Nvidia NGC version 22.03 (https://docs.nvidia.com/deeplearning/frameworks/pytorch-release-notes/rel_22-03.html). The experiments were performed from an NVIDIA DGX-2 server (16 GPU TESLA V100-SXM3-32GB) within Docker virtual machines.

5.8. **Figures**

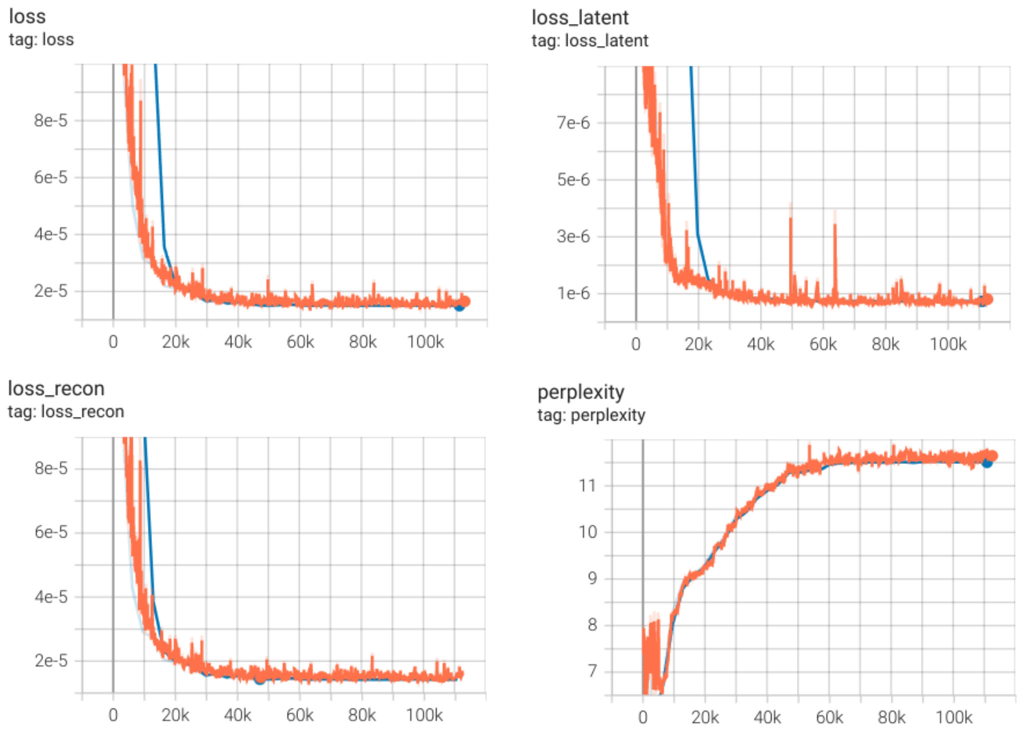

Figure S1: VQ-VAE sampled learning curves.

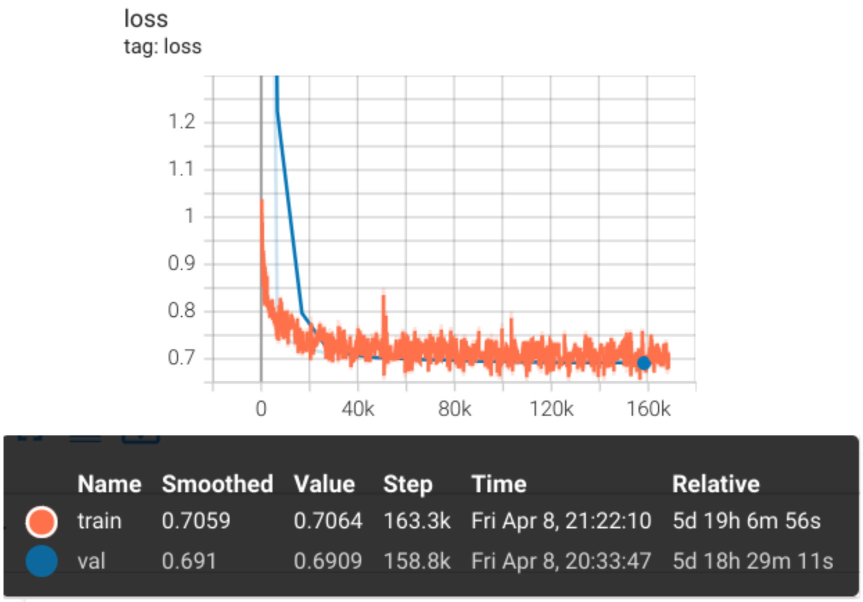

Figure S2: Transformer sampled learning curve.

5.9. **Tables**

Table S1: Statistical metrics for psychiatric symptoms in ABCD test set.

CBCL score	Brain region	*r*	*r p-value*
Total	Frontal_Sup_2_L	0.075	0.024
Total	Occipital_Sup_L	0.075	0.026

Total	Parietal_Sup_L	0.098	<0.001
Total	Parietal_Sup_R	0.081	0.004
Total	Precuneus_R	0.077	0.014
External	Frontal_Sup_2_L	0.075	0.022
External	Frontal_Sup_Medial_L	0.075	0.025
External	Parietal_Sup_L	0.089	<0.001
External	Parietal_Sup_R	0.079	0.008
Rule break	Frontal_Sup_2_L	0.076	0.016
Rule break	Frontal_Mid_2_R	0.078	0.010
Rule break	Frontal_Inf_Tri_R	0.074	0.036
Rule break	Frontal_Sup_Medial_L	0.080	0.006
Rule break	Parietal_Sup_L	0.087	0.001
Rule break	Parietal_Sup_R	0.080	0.006
Rule break	Precuneus_L	0.082	0.004
Conduct	Frontal_Sup_2_L	0.073	0.040
Conduct	Frontal_Sup_Medial_L	0.078	0.009
Conduct	Parietal_Sup_L	0.080	0.005
Aggressive	Parietal_Sup_L	0.082	0.003
Social	Precentral_R	0.074	0.034
Social	Frontal_Sup_2_L	0.084	0.002
Social	Frontal_Sup_2_R	0.080	0.006
Social	Frontal_Sup_Medial_L	0.074	0.031
Social	Frontal_Sup_Medial_R	0.081	0.005
Social	Calcarine_R	0.076	0.019
Social	Cuneus_L	0.081	0.004
Social	Cuneus_R	0.098	<0.001
Social	Occipital_Sup_L	0.091	<0.001
Social	Occipital_Sup_R	0.101	<0.001
Social	Occipital_Mid_L	0.073	0.047
Social	Occipital_Mid_R	0.081	0.004
Social	Postcentral_R	0.085	0.001
Social	Parietal_Sup_L	0.123	<0.001
Social	Parietal_Sup_R	0.102	<0.001
Social	Parietal_Inf_L	0.090	<0.001
Social	Angular_L	0.077	0.013
Social	Angular_R	0.085	0.001
Social	Precuneus_L	0.096	<0.001
Social	Precuneus_R	0.095	<0.001

*In column titles, **r** is Pearson's correlation between the group of symptoms and the likelihood of each brain region predicted by the neural network. Note: p-values are Bonferroni-corrected by the number of comparisons performed.*

Table S2: Statistical metrics for psychiatric symptoms in BHRCS.

CBCL score	Brain region	*r*	*r p-value*
Total	Vermis_3	-0.173	0.006
Total	Thal_VPL_L	0.180	0.002
Total	Red_N_L	0.162	0.027

Internal	Vermis_3	-0.182	0.002
Internal	Thal_VPL_L	0.171	0.009
Internal	VTA_L	0.164	0.020
Internal	Red_N_L	0.169	0.011
Somatic	Cerebellum_4_5_L	0.158	0.046
Somatic	Vermis_6	0.171	0.008
Somatic	Thal_VPL_L	0.169	0.011
Somatic	Red_N_L	0.161	0.031
Anxiety	Vermis_3	-0.160	0.034
Depression	Vermis_3	-0.160	0.033
Thought	Thal_VPL_L	0.179	0.003
Social	Vermis_3	-0.188	0.001
Social	Thal_VPL_L	0.163	0.025
Other	Thal_VPL_L	0.165	0.017
Other	Red_N_L	0.160	0.035

*In column titles, **r** is Pearson's correlation between the group of symptoms and the likelihood of each brain region predicted by the neural network. Note: p-values are Bonferroni-corrected by the number of comparisons performed.*

Table S3: Statistical metrics for ADHD evaluation in ADHD-200.

Symptom score	Brain region	*r*	*r p-value*
Inattentive	Frontal_Sup_2_L	-0.182	0.010
Inattentive	Frontal_Sup_2_R	-0.226	<0.001
Inattentive	Frontal_Sup_Medial_L	-0.192	0.003
Inattentive	Frontal_Sup_Medial_R	-0.231	0.000
Inattentive	OFCant_R	-0.176	0.021
Inattentive	Cerebellum_Crus2_L	-0.235	<0.001
Inattentive	Cerebellum_7b_L	-0.219	<0.001
Inattentive	ACC_sup_R	-0.171	0.035
Inattentive	SN_pc_R	-0.228	<0.001
Hyper impulsive	Frontal_Sup_2_L	-0.203	0.001
Hyper impulsive	Frontal_Sup_2_R	-0.266	<0.001
Hyper impulsive	Frontal_Sup_Medial_L	-0.229	<0.001
Hyper impulsive	Frontal_Sup_Medial_R	-0.266	<0.001
Hyper impulsive	Frontal_Med_Orb_L	-0.178	0.017
Hyper impulsive	Frontal_Med_Orb_R	-0.200	0.001
Hyper impulsive	Rectus_L	-0.185	0.008
Hyper impulsive	OFCant_R	-0.204	0.001
Hyper impulsive	Amygdala_R	-0.170	0.036
Hyper impulsive	Cerebellum_Crus1_L	-0.177	0.017
Hyper impulsive	Cerebellum_Crus2_L	-0.263	<0.001
Hyper impulsive	Cerebellum_Crus2_R	-0.187	0.006
Hyper impulsive	Cerebellum_3_L	-0.174	0.026
Hyper impulsive	Cerebellum_6_L	-0.177	0.017
Hyper impulsive	Cerebellum_7b_L	-0.248	<0.001
Hyper impulsive	Cerebellum_8_R	-0.191	0.004
Hyper impulsive	Thal_VA_R	0.181	0.012

Hyper impulsive	Thal_PuA_L	0.190	0.004
Hyper impulsive	ACC_pre_R	-0.182	0.011
Hyper impulsive	ACC_sup_R	-0.176	0.021
Hyper impulsive	SN_pc_R	-0.248	<0.001
Adhd index	SN_pc_R	-0.170	0.037

In column titles, ***r*** *is Pearson's correlation between the group of symptoms and the likelihood of each brain region predicted by the neural network. Note: p-values are Bonferroni-corrected by the number of comparisons performed.*

Table S4: Statistical metrics for ASD diagnosis in ABIDE-II.

Brain region	*auc*	*p-value*
Whole Brain	0.600	<0.001
Precentral_R	0.594	<0.001
Frontal_Inf_Oper_R	0.582	<0.001
Insula_L	0.578	<0.001
Cingulate_Mid_L	0.614	<0.001
Cingulate_Mid_R	0.584	<0.001
Cingulate_Post_L	0.572	<0.001
Hippocampus_L	0.597	<0.001
ParaHippocampal_L	0.627	<0.001
ParaHippocampal_R	0.576	<0.001
Calcarine_L	0.606	<0.001
Cuneus_L	0.580	<0.001
Cuneus_R	0.575	<0.001
Lingual_L	0.588	<0.001
Postcentral_L	0.599	<0.001
Postcentral_R	0.626	<0.001
Precuneus_L	0.627	<0.001
Paracentral_Lobule_L	0.582	<0.001
Paracentral_Lobule_R	0.604	<0.001
Putamen_L	0.591	<0.001
Heschl_R	0.620	<0.001
Temporal_Sup_L	0.616	<0.001
Temporal_Sup_R	0.609	<0.001
Temporal_Pole_Sup_L	0.605	<0.001
Temporal_Pole_Mid_R	0.594	<0.001
Cerebellum_Crus1_R	0.585	<0.001
Cerebellum_4_5_R	0.606	<0.001
Cerebellum_9_R	0.599	<0.001
Vermis_8	0.599	<0.001
Thal_AV_L	0.586	<0.001
Thal_PuM_L	0.603	<0.001
ACC_sup_L	0.587	<0.001
ACC_sup_R	0.593	<0.001
VTA_R	0.591	<0.001
SN_pc_R	0.569	<0.001

*In column titles, **auc** is the area under the receiver operation characteristic curve between the ASD diagnosis and the likelihood of each brain region predicted by the neural network. Note: p-values were calculated from permutation tests with 1000 iterations. After that, they were Bonferroni-corrected by the number of comparisons performed.*

Table S5: Statistical metrics of brain ageing.

Dataset	Brain region	*r*	*r p-value*
ABCD	Whole brain	-0.01	0.48
ABCD	Thal_VA_R	0.104	<0.001
ABCD	Thal_VL_R	0.075	0.023
ABCD	Red_N_L	0.076	0.014
BHRCS	Whole brain	-0.07	0.046
BHRCS	Pallidum_R	-0.195	<0.001
BHRCS	Cerebellum_4_5_R	-0.184	0.001
BHRCS	Cerebellum_6_R	-0.171	0.008
BHRCS	Vermis_1_2	-0.211	<0.001
BHRCS	Thal_IL_L	-0.194	<0.001
BHRCS	Thal_Re_L	-0.171	0.007
BHRCS	VTA_R	-0.163	0.023
ADHD-200	Whole brain	-0.19	<0.001
ADHD-200	Thal_PuI_L	-0.17	0.03
ABIDE-II	Whole brain	-0.33	<0.001
ABIDE-II	Precentral_L	-0.194	<0.001
ABIDE-II	Precentral_R	-0.27	<0.001
ABIDE-II	Frontal_Sup_2_R	-0.238	<0.001
ABIDE-II	Frontal_Mid_2_R	-0.15	0.048
ABIDE-II	Frontal_Inf_Oper_R	-0.224	<0.001
ABIDE-II	Rolandic_Oper_L	-0.217	<0.001
ABIDE-II	Rolandic_Oper_R	-0.234	<0.001
ABIDE-II	Supp_Motor_Area_L	-0.185	0.001
ABIDE-II	Supp_Motor_Area_R	0.212	<0.001
ABIDE-II	Frontal_Sup_Medial_L	-0.171	0.005
ABIDE-II	OFCant_L	-0.243	<0.001
ABIDE-II	OFCant_R	-0.309	<0.001
ABIDE-II	OFClat_L	-0.199	<0.001
ABIDE-II	OFClat_R	-0.166	0.01
ABIDE-II	Hippocampus_L	-0.226	<0.001
ABIDE-II	Cuneus_L	-0.158	0.021
ABIDE-II	Cuneus_R	-0.151	0.044
ABIDE-II	Angular_L	0.213	<0.001
ABIDE-II	Precuneus_L	-0.177	0.002
ABIDE-II	Paracentral_Lobule_L	-0.245	<0.001
ABIDE-II	Paracentral_Lobule_R	-0.153	0.033
ABIDE-II	Caudate_L	-0.293	<0.001
ABIDE-II	Caudate_R	-0.206	<0.001
ABIDE-II	Pallidum_L	0.225	<0.001
ABIDE-II	Heschl_R	-0.212	<0.001

ABIDE-II	Temporal_Sup_L	-0.165	0.01
ABIDE-II	Temporal_Sup_R	-0.18	0.002
ABIDE-II	Temporal_Pole_Sup_L	-0.189	<0.001
ABIDE-II	Temporal_Pole_Sup_R	-0.22	<0.001
ABIDE-II	Temporal_Pole_Mid_L	-0.186	0.001
ABIDE-II	Temporal_Pole_Mid_R	-0.337	<0.001
ABIDE-II	Temporal_Inf_R	-0.158	0.021
ABIDE-II	Cerebellum_Crus1_L	-0.276	<0.001
ABIDE-II	Cerebellum_Crus1_R	-0.369	<0.001
ABIDE-II	Cerebellum_Crus2_L	-0.252	<0.001
ABIDE-II	Cerebellum_Crus2_R	-0.337	<0.001
ABIDE-II	Cerebellum_3_L	-0.323	<0.001
ABIDE-II	Cerebellum_4_5_R	-0.3	<0.001
ABIDE-II	Cerebellum_6_L	-0.186	0.001
ABIDE-II	Cerebellum_6_R	-0.257	<0.001
ABIDE-II	Cerebellum_7b_L	-0.272	<0.001
ABIDE-II	Cerebellum_7b_R	-0.259	<0.001
ABIDE-II	Cerebellum_8_L	-0.191	<0.001
ABIDE-II	Cerebellum_8_R	-0.208	<0.001
ABIDE-II	Cerebellum_9_L	-0.283	<0.001
ABIDE-II	Cerebellum_9_R	-0.239	<0.001
ABIDE-II	Cerebellum_10_L	-0.279	<0.001
ABIDE-II	Cerebellum_10_R	-0.156	0.025
ABIDE-II	Vermis_1_2	-0.218	<0.001
ABIDE-II	Vermis_3	-0.192	<0.001
ABIDE-II	Vermis_8	-0.364	<0.001
ABIDE-II	Vermis_9	-0.186	0.001
ABIDE-II	Vermis_10	-0.264	<0.001
ABIDE-II	Thal_AV_L	-0.235	<0.001
ABIDE-II	Thal_VA_L	0.234	<0.001
ABIDE-II	Thal_VA_R	0.279	<0.001
ABIDE-II	Thal_VL_R	0.261	<0.001
ABIDE-II	Thal_IL_L	-0.194	<0.001
ABIDE-II	Thal_Re_R	-0.166	0.009
ABIDE-II	Thal_LGN_R	-0.189	<0.001
ABIDE-II	Thal_PuM_L	-0.352	<0.001
ABIDE-II	Thal_PuM_R	-0.25	<0.001
ABIDE-II	Thal_PuA_L	-0.27	<0.001
ABIDE-II	Thal_PuA_R	-0.212	<0.001
ABIDE-II	Thal_PuL_L	-0.28	<0.001
ABIDE-II	VTA_R	-0.357	<0.001
ABIDE-II	SN_pc_L	-0.235	<0.001
ABIDE-II	SN_pc_R	-0.337	<0.001
ABIDE-II	SN_pr_L	-0.271	<0.001
ABIDE-II	SN_pr_R	-0.213	<0.001
ABIDE-II	LC_L	-0.2	<0.001

ABIDE-II	LC_R	-0.294	<0.001

In column titles, ***r*** *is Pearson's correlation between the group of symptoms and the likelihood of each brain region predicted by the neural network. Note: p-values are Bonferroni-corrected by the number of comparisons performed.*

References

Ashburner, J., & Friston, K. J. (2000). Voxel-based morphometry - The methods. *NeuroImage*, *11*(6 I), 805–821. https://doi.org/10.1006/nimg.2000.0582

Mendes, S. L., Pinaya, W. H. L., Pan, P., & Sato, J. R. (2021). Estimating Gender and Age from Brain Structural MRI of Children and Adolescents: A 3D Convolutional Neural Network Multitask Learning Model. *Computational Intelligence and Neuroscience*, *2021*. https://doi.org/10.1155/2021/5550914

Penny, W., Friston, K., Ashburner, J., Kiebel, S., & Nichols, T. (2007). Statistical Parametric Mapping: The Analysis of Functional Brain Images. In *Statistical Parametric Mapping: The Analysis of Functional Brain Images*. Elsevier Ltd. https://doi.org/10.1016/B978-0-12-372560-8.X5000-1

Pinaya, W. H. L., Tudosiu, P. D., Gray, R., Rees, G., Nachev, P., Ourselin, S., & Cardoso, M. J. (2022). Unsupervised brain imaging 3D anomaly detection and segmentation with transformers. *Medical Image Analysis*, *79*, 102475. https://doi.org/10.1016/j.media.2022.102475

Rolls, E. T., Huang, C. C., Lin, C. P., Feng, J., & Joliot, M. (2020). Automated anatomical labelling atlas 3. *NeuroImage*, *206*. https://doi.org/10.1016/j.neuroimage.2019.116189

Sato, J. R., Biazoli, C. E., Salum, G. A., Gadelha, A., Crossley, N., Vieira, G., Zugman, A., Picon, F. A., Pan, P. M., Hoexter, M. Q., Anés, M., Moura, L. M., Del'Aquilla, M. A. G., Junior, E. A., McGuire, P., Rohde, L. A., Miguel, E. C., Bressan, R. A., & Jackowski, A. P. (2016). Connectome hubs at resting state in children and adolescents: Reproducibility and psychopathological correlation. *Developmental Cognitive Neuroscience*, *20*, 2–11. https://doi.org/10.1016/j.dcn.2016.05.002
